# Assessing the role of historical temperature regime and algal symbionts on the heat tolerance of coral juveniles

**DOI:** 10.1242/bio.047316

**Published:** 2020-01-23

**Authors:** K. M. Quigley, C. J. Randall, M. J. H. van Oppen, L. K. Bay

**Affiliations:** 1 Australian Institute of Marine Science, Townsville 4810, Australia; 2 Faculty of Science, The University of Melbourne, Victoria 3010, Australia

**Keywords:** Coral, Bleaching, Heat tolerance, Symbiodiniaceae, Selective breeding

## Abstract

The rate of coral reef degradation from climate change is accelerating and, as a consequence, a number of interventions to increase coral resilience and accelerate recovery are under consideration. *Acropora spathulata* coral colonies that survived mass bleaching in 2016 and 2017 were sourced from a bleaching-impacted and warmer northern reef on the Great Barrier Reef (GBR). These individuals were reproductively crossed with colonies collected from a recently bleached but historically cooler central GBR reef to produce pure and crossbred offspring groups (warm–warm, warm–cool and cool–warm). We tested whether corals from the warmer reef produced more thermally tolerant hybrid and purebred offspring compared with crosses produced with colonies sourced from the cooler reef and whether different symbiont taxa affect heat tolerance. Juveniles were infected with *Symbiodinium tridacnidorum*, *Cladocopium goreaui* and *Durusdinium trenchii* and survival, bleaching and growth were assessed at 27.5°C and 31°C. The contribution of host genetic background and symbiont identity varied across fitness traits. Offspring with either both or one parent from the northern population exhibited a 13- to 26-fold increase in survival odds relative to all other treatments where survival probability was significantly influenced by familial cross identity at 31°C but not 27.5°C (Kaplan–Meier *P=*0.001 versus 0.2). If in symbiosis with *D. trenchii*, a warm sire and cool dam provided the best odds of juvenile survival. Bleaching was predominantly driven by Symbiodiniaceae treatment, where juveniles hosting *D. trenchii* bleached significantly less than the other treatments at 31°C. The greatest overall fold-benefits in growth and survival at 31°C occurred in having at least one warm dam and in symbiosis with *D. trenchii*. Juveniles associated with *D. trenchii* grew the most at 31°C, but at 27.5°C, growth was fastest in juveniles associated with *C. goreaui*. In conclusion, selective breeding with warmer GBR corals in combination with algal symbiont manipulation can assist in increasing thermal tolerance on cooler but warming reefs. Such interventions have the potential to improve coral fitness in warming oceans.

This article has an associated First Person interview with the first author of the paper.

## Abstract (precis)

**Summary:** Survival of coral juveniles under heat stress can be significantly improved by breeding corals from historically warmer reefs with those from cooler reefs, and the provisioning with heat tolerant symbionts.

## INTRODUCTION

Coral reefs provide a suite of ecosystem services to people worldwide, including livelihoods, sustenance and storm protection (Moberg and Folke, 1999). The monetary contribution of reefs to regional economies is high (Young et al., 2012); the Great Barrier Reef (GBR) alone contributes 6.4 billion AUD to the Australian economy annually from tourism, fishing, recreation and scientific research, notwithstanding the benefits gained from its social, cultural and spiritual values (O'Mahoney et al., 2017). However, a range of local and global threats has had substantial negative impacts on the health and survival of corals. The GBR has seen long-term deterioration from crown-of-thorns starfish predation, cyclones and storms, water quality and climate change (De'ath et al., 2012). Marine heat waves in 2016 and 2017 resulted in extensive bleaching that severely impacted live coral cover across the northern and central sectors (Hughes et al., 2017). Temperatures that induce bleaching in corals, defined as the loss of their obligate dinoflagellate symbionts (family Symbiodiniaceae) and/or the reduction of symbiont pigments, are now three times more likely than they were three decades ago (Heron et al., 2016). Mitigation strategies are urgently needed to slow or halt further loss of corals from bleaching to maintain the ecological and social values of coral reefs until global warming is curbed.

Both host genetic background and Symbiodiniaceae identity influence the overall stress tolerance of the coral host and all of its microbial associates (i.e. the holobiont, Rohwer et al., 2002), and both have been implicated in the variation in survival from mass bleaching (Császár et al., 2009; Dixon et al., 2015; Hoadley et al., 2019; Manzello et al., 2019; Mieog et al., 2009). One study estimated that the adaptive potential of thermal tolerance in adult *Acropora millepora* corals is greater for the symbiont compared with the host, given high heritability in a number of key traits (Császár et al., 2010). The host genetic background also greatly influences stress tolerance, where having one or both parents from a warmer reef provided a 5-fold or 10-fold increase in survival at high temperatures of coral larvae (Dixon et al., 2015), and demonstrates a strong link between host genotype and thermal tolerance. Finally, an increased potential for a dominant role of the host to confer thermal tolerance has been implicated in cases where colonies are collected from areas with more extreme thermal histories (Dixon et al., 2015; Thompson and van Woesik, 2009).

Coral photosymbionts within the family Symbiodiniaceae exhibit a high level of trait variability and local adaptation (Howells et al., 2012; LaJeunesse et al., 2018; reviewed in Quigley et al., 2018). Symbiodiniaceae diversity *in hospite* also greatly influences coral bleaching tolerance (McIlroy et al., 2016; Mies et al., 2018; Yuyama et al., 2016). For example, a change in relative abundance (shuffling) from *Cladocopium* to *Durusdinium* increased bleaching tolerance of adult colonies by up to 1.5°C (Berkelmans and van Oppen, 2006), in which the presence of *Durusdinium* explained ∼24% of the variability in bleaching (Baird et al., 2009; Mizerek et al., 2018). Symbiodiniaceae shuffling during bleaching events can also occur in juveniles (Yorifuji et al., 2017; Yuyama and Higuchi, 2014). *Acropora tenuis* juveniles harbouring *Cladocopium goreaui* experienced greater mortality compared to those with *Durusdinium* at elevated temperature and light levels (Yuyama et al., 2016), although the opposite pattern has also been found (Abrego et al., 2008). Juveniles with mixed communities of *Symbiodinium tridacnidorum*,* C. goreaui* and *D. trenchii* exhibited increased survival at 30°C compared to 31–32°C, with surviving juveniles harbouring more *D. trenchii* (Yorifuji et al., 2017). This indicates the importance of different symbionts in determining host temperature tolerance.

Trade-offs in coral holobiont traits exist for various coral-Symbiodiniaceae associations, especially between *Cladocopium* and *Durusdinium* in both juveniles (Cantin et al., 2009; Little et al., 2004) and adults (Jones and Berkelmans, 2010). Corals hosting *Durusdinium* often survive better at high temperatures compared to those with *Cladocopium,* but grow slower at lower temperatures (Cantin et al., 2009; Cunning et al., 2015; Jones and Berkelmans, 2010; Little et al., 2004). Moreover, juveniles harbouring *S. microadriaticum* grew faster than those with *Breviolum minutum* (McIlroy and Coffroth, 2017), and skeletal growth was faster in juveniles with *C. goreaui* compared with *Durusdinium* despite lower *C. goreaui* population growth rates (Yuyama and Higuchi, 2014). Host-driven variation in thermal tolerance (Baird et al., 2009; Cunning et al., 2015) has rarely been examined in concert with Symbiodiniaceae identity or been manipulated to experimentally quantify changes in thermal tolerance due to the host-symbiont interaction (Abrego et al., 2008; Kenkel et al., 2015b; Manzello et al., 2019; Mieog et al., 2009).

To address this research gap, we crossed coral colonies sourced from a comparatively warm northern reef of the GBR that had survived both the 2016 and 2017 mass coral bleaching events with colonies from a central reef that experienced lower mean and maximum annual temperatures and also survived the 2016 and 2017 bleaching events. Juveniles from these crosses were infected with one of three Symbiodiniaceae taxa (*S. tridacnidorum*, *C. goreaui* and *D. trenchii*) and survival, growth, and bleaching were assessed at two temperatures (27.5°C and 31°C). The interactive effects of host genotype and symbiont identity on juvenile coral performance was then estimated for three key fitness traits; survival, bleaching tolerance, and growth.

## RESULTS

**Fig. 1.**
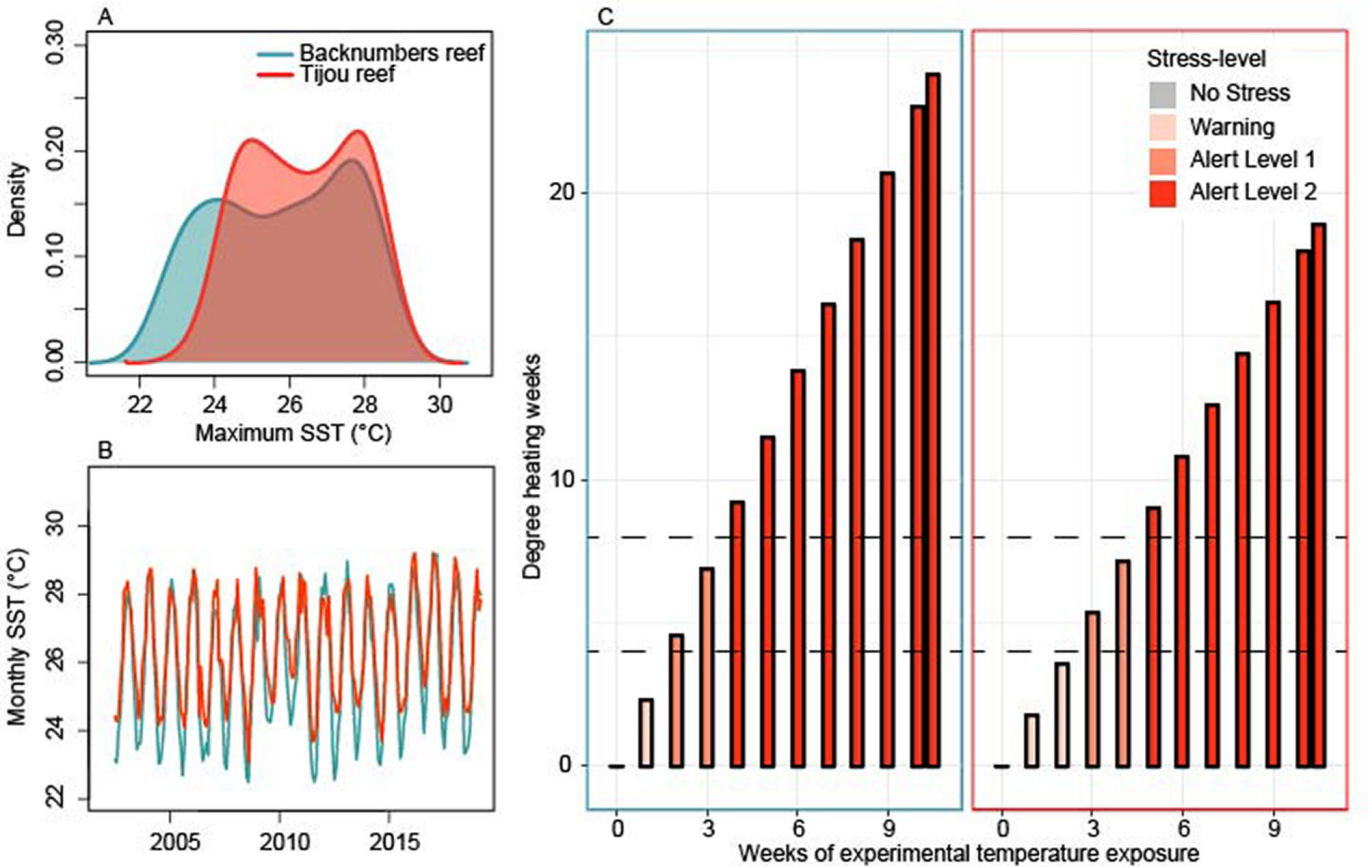
(A,B) Mean monthly sea-surface temperature (°C) records for Backnumbers reef (central GBR) and Tijou reef (far northern GBR) from June 2002 through March 2019 from the Aqua MODIS satellite. (C) Estimated level of experimental thermal stress experienced in the 31°C treatment for juveniles sourced from Backnumbers (blue outlined box) and Tijou (red outlined box) reefs. Barplot colours represent NOAA CoralReefWatch Status Alert Categories (grey to red). Horizontal dashed lines represent thresholds when DHW>4 (Alert Level 1) or >8 (Alert Level 2). NOAA Coral Reef Watch Bleaching Alert System values were used to determine the degree heating weeks and alert levels for each coral reef.

Host genetic background at the familial cross (WW1, WW2, WW3, WC, CW) and parental source (WW, WC, CW; W indicates warm far northern parent and C indicates cool central parent) levels influenced juvenile survival, growth and bleaching across the three symbiont treatments at 27.5°C and 31°C (Fig. 1, Table 1). Juveniles with two warm parents generally demonstrated overall higher performance across fitness traits (detailed below), whilst juveniles with a warm dam also performed better across some fitness traits. Secondly, symbiont treatment influenced juvenile survival and growth under thermal stress, with juveniles hosting *D. trenchii* generally performing better than the other symbionts across all familial crosses. This pattern was particularly strong for bleaching fitness in juveniles of parents sourced from the warm reef.

**Table 1.**
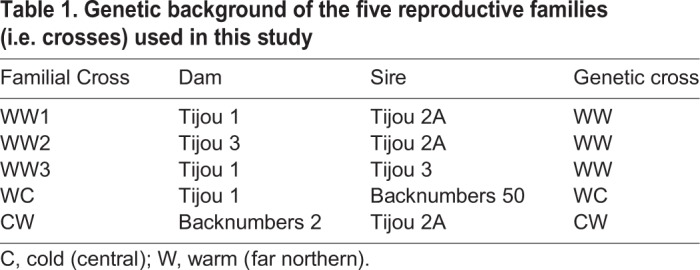
**Genetic background of the five reproductive families (i.e. crosses) used in this study**

**Fig. 2.**
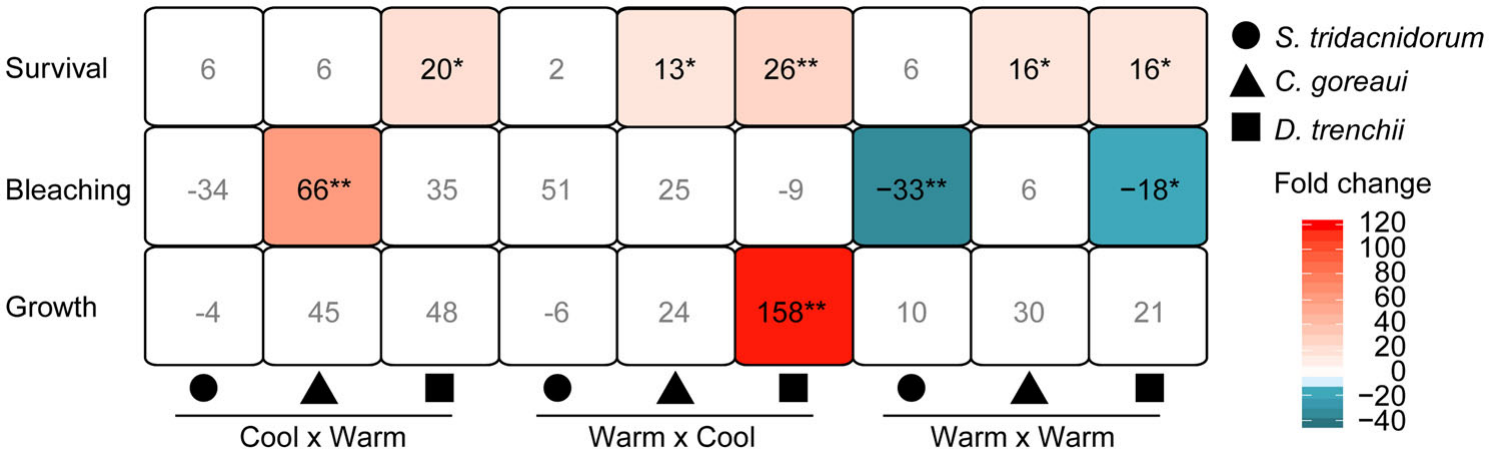
**Significant fold changes in the log-odds in survival and changes in growth and bleaching between Time_initial_** **and Time_final_** **due to genetic background and symbiont combinations grouped by treatment (Cool×Warm, Warm×Cool or Warm×Warm) and symbiont type [*S. tridacnidorum*,** ***C. goreaui* and *D. trenchii* (yellow, blue or red)].** Negative values represent decreased log-odds of bleaching (i.e. less bleaching). A separate model was fit for each trait. White boxes represent non-significant *P*-values (GLMM or MCMCglmm), all coloured boxes are significant (*P<0.05, **P<0.005, exact values are given in the text). Significance values are were calculated for each treatment group relative to the global mean of all treatments (i.e. the mean of all other treatments together) averaged across both temperature treatments.

Hosting *D. trenchii*, regardless of host genetic background, conferred significantly higher odds of survival in juveniles, including up to a 26-fold increase in survival odds relative to all other treatments [i.e. in comparison to the global mean; generalized linear mixed model (GLMM) WC *P=*0.002] (Fig. 2) and 20-fold increase in juveniles with a warm sire and *D. trenchii* (GLMM *P=*0.02). Juveniles with two warm parents and either *C. goreaui* or *D. trenchii* displayed a 16-fold increase in the odds of survival (GLMM both *P=*0.01). Juveniles with one warm dam and *C. goreaui* exhibited a 13-fold increase in survival (GLMM *P=*0.03). Odds of survival were not significantly greater for juveniles with a cool dam hosting *C. goreaui* (GLMM *P=*0.2) or any of the genetic crosses with *S. tridacnidorum* (GLMM *P=*0.2-0.7).

Familial cross and the source of the dam were also important in determining other fitness traits at elevated temperatures. Juveniles with two warm parents were the only genetic combinations with increased log odds of bleaching resistance in surviving juveniles (Fig. 2). These juveniles, hosting *D. trenchii* or *S. tridacnidorum*, exhibited an 18–33-fold decrease in probability of bleaching, respectively [Markov chain Monte Carlo (MCMC)_glmm_: *D. trenchii P*=0.008; *S. tridacnidorum P*=0.004], whereas juveniles with one cool dam and *C. goreaui* exhibited 66-fold increased odds of bleaching (MCMC_glmm_ *P*=5e-4). Only juveniles produced from a warm dam significantly conferred increased odds of growth, but only when juveniles were associated with *D. trenchii* (MCMC_glmm_ *P=*0.001) (Fig. 2).

### Temperature and symbiont effects on survival, growth and bleaching

#### Survival

After 70 days, overall juvenile survival at 27.5°C was higher than at 31°C (47±0.03% versus 21±0.03%). Symbiont identity explained 4.4% of model variation in survival, whereas familial cross identity explained 0.7% and temperature explained 11.7% (Table 2).

**Table 2.**
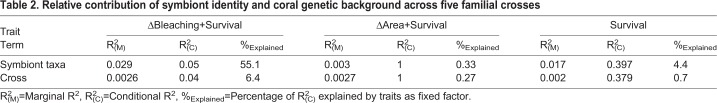
**Relative contribution of symbiont identity and coral genetic background across five familial crosses**

**Fig. 3.**
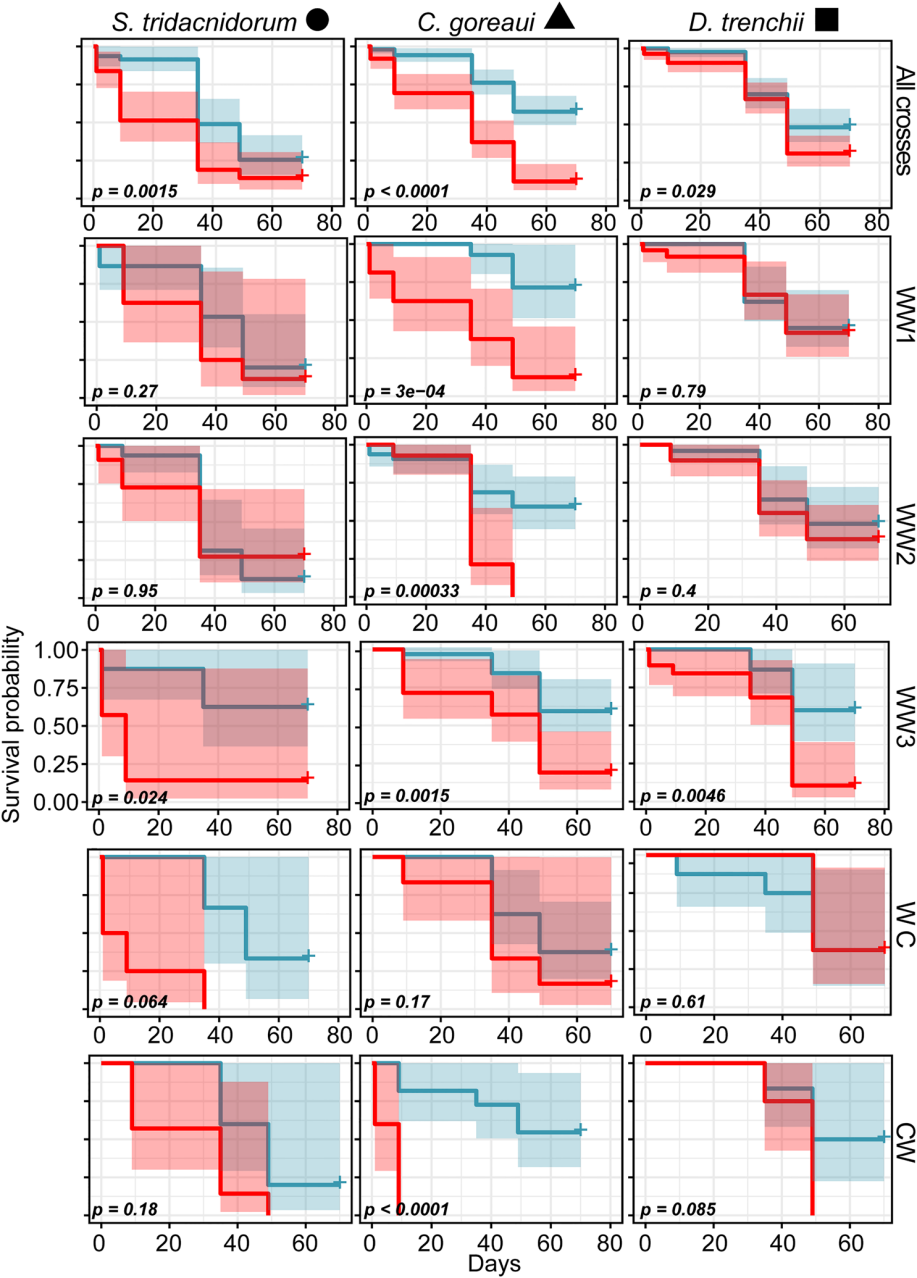
**Kaplan–Meier survival probabilities of juveniles and associated *P*-values across Symbiodiniaceae treatments and distinct familial cross identities 27.5°C (blue) and 31°C (red).** The top row corresponds to survival averaged across all families for each symbiont taxa.

Survival varied significantly across juveniles with distinct familial cross identities and symbionts (Fig. 3). Juveniles exposed to different Symbiodiniaceae all exhibited significantly higher probability of survival at 27.5°C compared to at 31°C [mean survival at 31°C at the final timepoint±s.e.: *D. trenchii*: 45.7±5% versus 30.5±5%; Kaplan–Meier (KM) comparison between 27.5°C and 31°C *P*=0.029; *C. goreaui*: 56.7±5% versus 12.9±4%; KM *P*<0.0001; *S. tridacnidorum*: 26.5±6% versus 13±5%; KM *P*=0.0015]. The three top surviving familial crosses at 31°C were those with *D. trenchii* symbionts with at least one warm dam (WC, WW1, WW2: 50±18% to 38±9%, Fig. 3).

Juvenile survival varied significantly amongst comparisons across all familial cross identities and symbionts at 27.5°C (KM *P=*0.008) and 31°C (KM *P<*0.0001). However, when averaged over Symbiodiniaceae treatments, there was a significant difference in survival probability due to familial cross at 31°C (KM *P=*0.0019*)*, but not at 27.5°C (KM *P=*0.2). This was predominantly driven by the poor performance at 31°C of CW when associated with *C. goreaui* (KM *P<*0.0001), and across familial crosses exposed to *S. tridacnidorum* generally (KM *P=*0.056).

The three crosses of juveniles with two warm parents exhibited differential survival between the two temperature treatments infected with the three symbiont taxa (Fig. 3). For example, juveniles from the WW1 and WW2 familial crosses infected with *D. trenchii* did not differ in survival probabilities between 27.5°C and 31°C, although WW3 juveniles did (KM *P=*0.79, 0.4 and 0.0046, Fig. 3). All juveniles infected with *C. goreaui* survived significantly less at 31°C (KM *P=*3e-4-0.0015, Fig. 3). Juveniles with one warm dam survived equally well at 31°C compared to 27.5°C when exposed to *C. goreaui* and *D. trenchii* (KM *P=*0.17–0.61, Fig. 3). Juveniles at 31°C with one cool dam exhibited the lowest survival over the shortest period of time, particularly when hosting *C. goreaui* (KM *P<*0.0001, Fig. 3). Survival was not significantly worse for the other two symbiont taxa across the two temperatures (KM *P=*0.18 and 0.085, Fig. 3).

#### Growth

##### Growth and mortality

**Fig. 4.**
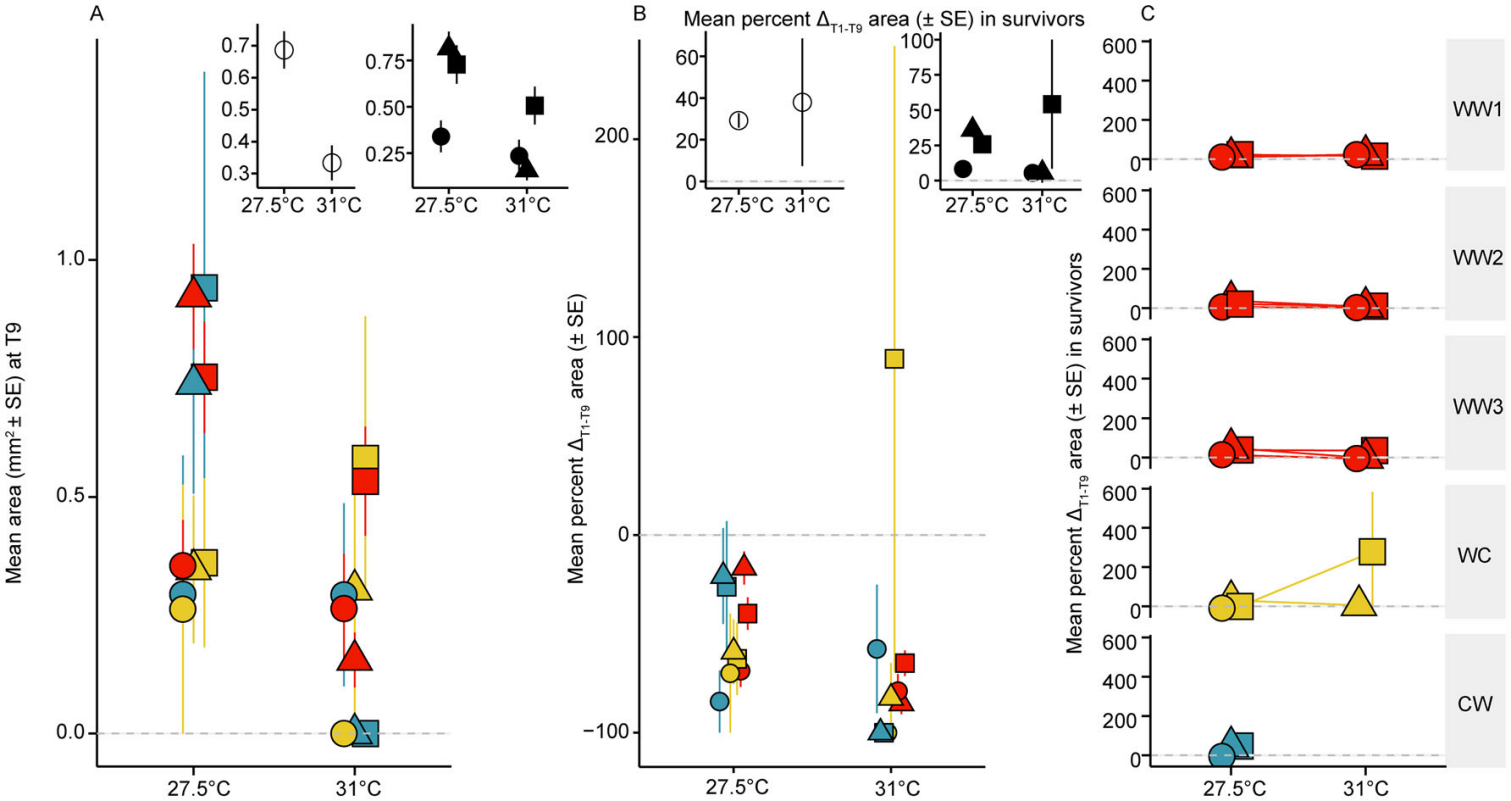
**Juvenile growth.** (A) Mean area of juveniles (mm^2^±s.e.) at the final timepoint (70 days, T9) across genetic backgrounds (red: Warm×Warm, yellow: Warm×Cool, blue: Cool×Warm). Insets in A show mean area of juveniles (mm^2^±s.e.) at the final timepoint across all treatments (left) and mean area of juveniles (mm^2^±s.e.) at the final timepoint across three symbiont treatments (right) (circles: *S. tridacnidorum*, triangles: *C. goreaui*, squares: *D. trenchii*). (B) Growth (percent change in area, mm^2^±s.e. from Time_initial_ to Time_final_) of juveniles at 27.5°C and 31°C with different genetic backgrounds and symbiont treatments. Insets in B show percent change in area (mm^2^±s.e. from Time_initial_ to Time_final_) of only surviving juveniles averaged across 27.5°C and 31°C and by symbiont treatments. (C) Growth (percent change in area, mm^2^±s.e. from Time_initial_ to Time_final_) of only surviving juveniles at 27.5°C and 31°C across familial crosses and symbiont treatments. Panels represent values that include juvenile growth and mortality (see Materials and Methods for further explanation) unless otherwise stated by ‘in survivors’ (insets in B and all panels in C).

Familial cross and symbiont identity explained little of the model variation in growth and mortality between the two temperature treatments (0.27% and 0.33%, respectively) (Table 2). By the final timepoint, juveniles at 27.5°C were on average two times larger compared to juveniles at 31°C (0.7±0.06–0.3±0.05 mm^2^, Fig. 4A insets). Juveniles at 27.5°C with *C. goreaui* and *D. trenchii* were the largest, and all juveniles decreased in size at 31°C, in which juveniles infected with *C. goreaui* were the smallest overall. At 27.5°C, juveniles with one warm dam and a cool sire were generally smaller (Fig. 4A). At 31°C, juveniles with at least one warm dam were on average larger, especially when infected with *D. trenchii* (0.6±0.11–0.5±0.29 mm^2^).

##### Growth of surviving juveniles

If only juveniles that survived were assessed, symbiont identity explained 69.8% of the variability in the change in growth, and genetic background only explained 3.8% (Table 2). Overall, from the first to last timepoint, all juveniles at 27.5°C and 31°C decreased in size, with the exception of juveniles with a warm dam and *D. trenchii* symbionts (+88.9±158%; Fig. 4B). Examining only those juveniles that survived to 70 days shows they grew in both temperature treatments (>20–40% change, Fig. 4B inset). Surviving juveniles with *D. trenchii* grew more compared to juveniles hosting *S. tridacnidorum* and *C. goreaui* at 31°C compared to 27°C, although these differences were not significant, likely due to the high variability across surviving juveniles with *D. trenchii* (Tukey post-hoc Gaussian MCMC_GLMM_ *P=*0.5–0.8) (Fig. 4B inset). When infected with *S. tridacnidorum*, the few surviving juveniles in all five crosses exhibited negative to moderate growth regardless of host genetic background at both temperatures (27.5°C: −9.5–13.6%; 31°C: −5–23%) (Fig. 4C). Surviving juveniles infected with *C. goreaui* grew more compared to juveniles with *D. trenchii* and *S. tridacnidorum* at 27.5°C across the five crosses, in which the greatest average growth occurred at 27.5°C (10.6–47.9%), compared with 31°C (−2.4–21%). There were no significant differences in growth between 27.5°C and 31°C when averaged across familial crosses with *S. tridacnidorum* (Tukey post-hoc Gaussian MCMC_GLMM_, *P=*0.86–0.98) or *C. goreaui* (*P=*0.12–0.92) or *D. trenchii* (*P=*0.13–0.99).

All but two *D. trenchii* crosses at 27.5°C and 31°C increased in size (13.5–277.8% change) over the 70-day experiment (exceptions: WC at 27°C, –0.3%; CW at 31°C, no survivors) (Fig. 4C). This is in comparison to mean percent change in growth that varied the most between juveniles at 31°C when infected with *C. goreaui* (Fig. 4B). Across temperatures, there were no significant differences in growth in any pairwise comparisons across the five crosses with surviving juveniles (Tukey post-hoc Gaussian MCMC_GLMM_, *P=*0.34–1) or within each cross (*P=*0.07–0.1) after averaging across symbiont identity.

#### Bleaching and mortality

**Fig. 5.**
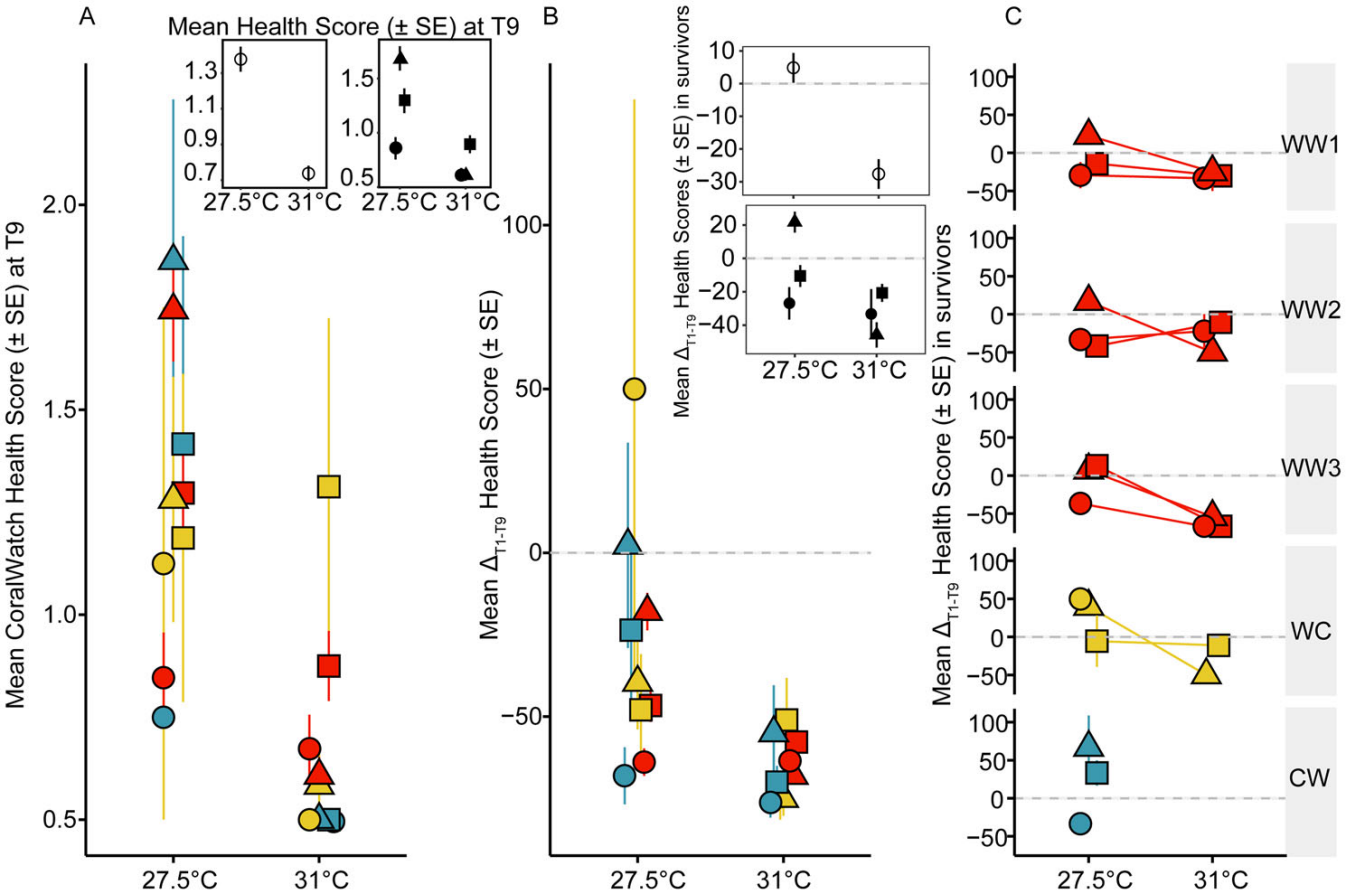
**Juvenile bleaching.** (A) Mean CoralWatch Health Score of juveniles (±s.e.) at the final timepoint (70 days, T9) across genetic backgrounds (red: Warm×Warm, yellow: Warm×Cool, blue: Cool×Warm). Insets in A show mean CoralWatch Health Score of juveniles (±s.e.) at the final timepoint across all treatments (left) and mean CoralWatch Health Score of juveniles (mm^2^±s.e.) at the final timepoint across three symbiont treatments (right) (circles: *S. tridacnidorum*, triangles: *C. goreaui*, squares: *D. trenchii*). (B) Bleaching (percent change in mean CoralWatch Health Score±s.e. from Time_initial_ to Time_final_) of juveniles at 27.5°C and 31°C with different genetic backgrounds and symbiont treatments. Insets in B show percent change in bleaching (percent change in mean CoralWatch Health Score±s.e. from Time_initial_ to Time_final_) of only surviving juveniles averaged across 27.5°C and 31°C and by symbiont treatments. (C) Bleaching (percent change in mean CoralWatch Health Score±s.e. from Time_initial_ to Time_final_) of only surviving juveniles at 27.5°C and 31°C across familial crosses and symbiont treatments. Panels represent values that include the Health Score and mortality (see Materials and Methods for further explanation) unless otherwise stated by ‘in survivors’ (insets in B and all panels in C).

After 70 days, juveniles at 27.5°C scored greater than 1.3 (less bleached) on the CoralWatch Health Score scale, whereas juveniles at 31°C scored less than 0.7 (more bleached) (Fig. 5A inset). Symbiont identity explained most of the model variation between the two temperature treatments (55.1%) in the change in Health Scores and mortality compared with the genetic background of the coral juveniles (6.4%) (Table 2).

Juveniles with *C. goreaui* and *D. trenchii* exhibited the darkest colouration at 27.5°C and 31°C, respectively, at the final timepoint (Fig. 5A inset). At 27.5°C, Health Scores of juveniles with different genetic backgrounds were generally distributed by symbiont type, with the darkest colouration measured for juveniles with *C. goreaui*, then *D. trenchii* and finally *S. tridacnidorum* (Fig. 5A). At 31°C, juveniles with two warm parents exhibited on average the highest Health Scores and those with a cool parent exhibited the lowest (Fig. 5A). However, juveniles with one warm and one cool parent were the darkest at 31°C, (WC 1.3±0.6), WW2 and WW1 intermediate (0.82±0.2 and 0.5±0.15 respectively) and CW and WW3 bleached heavily (0.05±0.05) (data not shown).

At 27.5°C, juveniles with one warm dam and one cool sire associated with *S. tridacnidorum* increased their Health Score the most from the first to last timepoint (∼50%), whereas Scores decreased the most in juveniles with either one warm dam or both warm parents with *S. tridacnidorum* (Fig. 5B). Alternatively, at 31°C, on average all juveniles from all genetic crosses decreased in their Health Scores, where juveniles with one warm or cool dam varied in their temperature response (Fig. 2B).

##### Bleaching in surviving juveniles

Overall, after 70 days, surviving juveniles at 27.5°C darkened in their Health Scores (+5% change), whereas juveniles at 31°C paled (−28% change) (Fig. 5B inset). In surviving juveniles compared across models, symbiont identity explained 92.9% of the variability in bleaching and genetic background explained 64.8%.

Surviving juveniles infected with *C. goreaui* darkened at 27.5°C (+22%), but bleached the most at 31°C (−46%) (Fig. 5B inset). Juveniles infected with *D. trenchii* paled at 27.5°C (−11%), but bleached less at 31°C (−21%) than juveniles associated with other Symbiodiniaceae. Surviving juveniles infected with *S. tridacnidorum* paled at both temperatures in four of the five crosses (−26.9 to −33.3 and −66 to +50%) (Fig. 5B inset, C). Health Scores in the surviving juveniles in the 31°C treatment ranged from −11% change in colouration (WW2 with *D. trenchii*) to −67% (WW3 with *S. tridacnidorum* and *D. trenchii*), indicating substantial phenotypic variability for bleaching tolerance within the warm parent crosses (Fig. 5C). WW2 juveniles bleached the least at 31°C compared to other familial crosses when averaged over all symbiont combinations (−16±7%) (Fig. 5C). The other two warm parent crosses bleached at 31°C across all symbiont treatments (WW1: −29±26%, WW3: −58±6%). At 31°C, WC juveniles with *D. trenchii* remained relatively unchanged in their Health Scores compared with the 27.5°C treatment (Fig. 5C). WW2 juveniles bleached the least compared to other treatments when infected with *D. trenchii* at 31°C compared with 27.5°C (Fig. 5C).

Irrespective of symbiont identity, bleaching in WW2 juveniles was only significantly less compared to juveniles from WW3 (Tukey post-hoc Gaussian GLMM, *P=*0.037). There were no significant differences in bleaching alone amongst the other WW crosses (Tukey post-hoc Gaussian GLMM, *P=*0.995–1) or WC (*P=*0.76–1).

## DISCUSSION

### Variable contribution of symbiont and host genetics across multiple coral traits

Symbiont identity and host genetic background varied in their influence on juvenile fitness. Bleaching was significantly reduced in surviving offspring with two warm parents, with the lowest bleaching tolerance predicted by a cool dam. Compared to bleaching, growth was less influenced by both host genetic background and symbiont identity, although juveniles hosting *D. trenchii* with a warm dam grew the most at 31°C. Having a dam or both parents sourced from a warmer reef significantly improved juvenile survival at 31°C by up to 26-fold, irrespective of whether juveniles hosted *D. trenchii* or *C. goreaui*. Our findings show that the heat tolerance boost to larvae provided by parents sourced from a warm reef, which was previously demonstrated in larvae (Dixon et al., 2015), is maintained at the juvenile life-history stage. Interestingly, under field conditions, the contribution of host genetics was found to be minimal in the Caribbean species *Porites astreoides* (but see Kenkel et al., 2015b), whereas symbiont identity and environmental factors explained variation in growth, survival and thermal stress in *Acropora millepora* on the GBR (Mieog et al., 2009). Hence, these results demonstrate the role of parental genotype in increasing juvenile survival in a broadcast spawning species under elevated temperature.

### Survivors from reefs that bleached with historically warmer temperatures provide increased survival benefits to offspring

When averaged over Symbiodiniaceae treatments, juvenile survival was significantly influenced by familial cross at 31°C but not at 27.5°C, where juveniles with at least one warm dam exhibited higher survival probability at warmer temperatures. Warmer days and variable environmental conditions may select for genotypes that are more able to cope with increasing sea surface temperatures (Palumbi et al., 2014). Therefore, the warmer conditions in the far northern GBR suggest that the surviving corals on these reefs may harbour the greatest frequency of adaptive genetic variants associated with thermal tolerance (Dixon et al., 2015; Jin et al., 2016). Our results demonstrate that having at least one parent, but especially both, from a warmer reef and as bleaching survivors provides a 16–26-fold increase in odds of overall juvenile survival. Furthermore, if paired with *D. trenchii*, a warm sire and cool dam provides the best odds of juvenile survival. Compared to previous estimates of a 5–10-fold increase in survival of aposymbiotic *A. millepora* larvae (Dixon et al., 2015), having a bleaching-surviving parent who is also sourced from a warmer reef represents a 16-fold greater thermal tolerance boost, although differences in gamete developmental temperatures between these two populations should also be considered. Physiological performance at ambient temperatures should also be considered given the variable breadth of mean monthly temperatures across reefs, for example, the wider temperature range of Backnumbers reef compared to Tijou in winter. The benefit of using survivors from reefs with historically warmer environments to enhance juvenile survival therefore appears to be promising but requires further testing.

### Host–symbiont interactions influence juvenile fitness in response to thermal stress

The greatest overall fold-benefits in survival and growth under elevated temperature occurred in having at least one warm dam and in symbiosis with *D. trenchii*. There was also a trend towards higher probability of survival in juveniles with *D. trenchii* compared with *C. goreaui* when exposed to warmer but not ambient temperatures, consistent with previous reports in adult corals (Baker et al., 2004; Berkelmans and van Oppen, 2006) and *A. millepora* juveniles (Mieog et al., 2009). Interestingly, *D. trenchii* provided little increased survival odds at 31°C compared with *C. goreaui* in juveniles with two warm parents.

Survival at 31°C also varied significantly across the different juvenile crosses infected with *C. goreaui*, where juveniles produced from a single cool dam crossed with a warm sire fared the worst, although direct effects of seawater temperature during gamete development may also influence these patterns in survival. These results add further complexity to understanding how host–symbiont interactions relate to thermal tolerance, including at the coral species-level (Abrego et al., 2008; Mieog et al., 2009), across developmental stages and now by different host genotypes and symbiont taxa, which can account for 91% of bleaching variance (Hoadley et al., 2019). These results may indicate maternal effects that extend into the juvenile phase or that coral genotype-symbiont interactions are heritable traits (Parkinson and Baums, 2014; Quigley et al., 2016), and appear to be predominantly driven by host genotypic differences between crosses.

We also detected high variability in survival, bleaching and growth across the different crosses with two warm parents. Given this variability, the high heritability of many of these traits, and the strong influence of host genotype (Cunning et al., 2015; Drury et al., 2017; Kenkel et al., 2013, 2015b), caution should be taken when choosing source material for brood stock production during selective breeding. The selection of parental genotypes from bleaching survivors from naturally warm far northern reefs may require substantial effort given the high variability in warm parental genotypes seen here, but when identified, should greatly increase the chances of producing heat tolerant individuals for reef restoration.

### Trade-offs between survival and growth across different symbiont associations

Hosting *Durusdinium* at elevated temperatures generally provides a significant increase in heat tolerance, but decreased host growth compared with hosting *Cladocopium* (Jones and Berkelmans, 2010; Stat and Gates, 2011; but see Howells et al., 2013). We found evidence of trade-offs between survival and growth in which juveniles with *D. trenchii* grew more and had greater probability of survival at elevated temperatures whereas juveniles infected with *C. goreaui* grew more at 27.5°C. This aligns with previous reports for a growth advantage of corals hosting *C. goreaui* at cooler temperatures (Cantin et al., 2009; Little et al., 2004). Increased growth rates in juveniles with *Durusdinium* have been observed and may be associated with these symbionts colonizing coral juveniles at faster rates compared to *C. goreaui* (Yuyama and Higuchi, 2014) given initial levels of symbiosis establishment and/or pigmentation varies by symbiont type (Cumbo and van Oppen, 2018; Yuyama and Higuchi, 2014). However, we only saw slight differences in symbiosis establishment across symbiont treatments at the start of the experiment, where coral colouration is a proxy for cell density (Mean CoralWatch Health Scores at day 1: *C. goreaui*, 2.1; *S. tridacnidorum*, 2.4; *D. trenchii*, 2.5).

We found no evidence for a trade-off in growth and survival for *Durusdinium* at 31°C, where juveniles hosting *Durusdinium* would have been expected to have greater probability of survival at warmer temperatures but grow less (Pettay et al., 2015; but see Manzello et al., 2019). Modelling results suggest that these trade-offs may detrimentally impact reef recovery as the increased abundance of heat tolerant *D. trenchii* leads to reductions in host growth rates (Ortiz et al., 2013). However, juveniles hosting *D. trenchii* both exhibited higher probability of survival and grew more compared to juveniles hosting other Symbiodiniaceae taxa at warmer temperatures. This aligns with previous evidence showing no trade-off costs between growth and survival for *Durusdinium* once temperatures increase past 26°C (Cunning et al., 2015).

The strong symbiont effect found here was surprising given the initially low Symbiodiniaceae CoralWatch Health Score (i.e. Score<3≈10^6^ cells/cm^2^, Siebeck et al., 2006). Symbiodiniaceae cell density strongly influences host physiology, and if the total cost to the host in maintaining symbionts is low and the benefit high, the optimal symbiont density may be low (Cunning and Baker 2014). Hence, even low densities of symbionts may significantly influence host functioning, as our data suggest. Indeed, after only 4 h post-exposure to Symbiodiniaceae, small but significant host transcriptional responses co-occurred with symbiont uptake in coral juveniles (Mohamed et al., 2016) and with as few as four Symbiodiniaceae cells in *Aiptasia* larvae (Bucher et al., 2016). These data suggest that even at low densities, symbionts may significantly influence host growth and survival.

### Drivers of bleaching tolerance in coral juveniles

Bleaching responses were variable across symbiont treatments at elevated temperatures, consistent with previous reports (reviewed in Quigley et al., 2018). Although initial infection across temperatures and symbiont treatments were similar (see Materials and Methods), variability in bleaching responses at later time points may have been due to differences in symbiosis establishment rates and/or chlorophyll content (Cumbo et al., 2018; Yamashita et al., 2014; Yuyama and Higuchi, 2014). At elevated temperatures, juveniles harbouring *D. trenchii* bleached less compared to juveniles hosting *S. tridacnidorum* and *C. goreaui*. Taken in conjunction with our results for survival and growth, this suggests that the thermal optimum for the coral–*D. trenchii* association is higher compared to other combinations.

Although juveniles did not exhibit bleaching (i.e. a negative change in Health Score) at 27.5°C when averaged among all genetic backgrounds and symbiont treatments, juveniles infected with *S. tridacnidorum* and *D. trenchii* paled or bleached at 27.5°C. Juveniles with *S. tridacnidorum* also bleached at 31°C. This may indicate that the optimal temperature for *D. trenchii* is higher than 27.5°C. It may also suggest that the symbiosis between *A. spathulata* juveniles and *S. tridacnidorum* was not stable (*sensu* van Oppen et al., 2001) or compatible, an observation that has been made for multiple types within *Cladocopium* and *S. microadriaticum* (Mieog et al., 2009). *S. tridacnidorum* is a key early symbiont partner for some *Acropora* juveniles (Quigley et al., 2016; Suzuki et al., 2013) but is lost at later life stages (Quigley et al., 2017b). Thus far, only ITS1-type C2 (*sensu* van Oppen et al., 2001, equivalent to ITS2-type C3 *sensu* LaJeunesse, 2002) has been identified from adult *A. spathulata* collected from Pelorus and Heron Islands in the central and southern GBR, respectively (Genbank accession AF380538, van Oppen et al., 2001), suggesting that over time, strains within *Symbiodinium* do not provide equivalent fitness benefits across ontogeny.

### Signatures of local adaption

Evidence of local adaptation fuelling fitness trade-offs is well known (Hereford, 2009), and the trade-off costs for immigrants are high in some reef environments (Howells et al., 2013). For example, adult fragments translocated between inshore and offshore sites in the Florida Keys exhibited high survival but reduced growth (Kenkel et al., 2015a) and colonies with *Cladocopium* versus *Durusdinium* had higher egg densities in the absence of temperature anomalies (Jones and Berkelmans, 2011). Intraspecific hybrid juveniles produced from parents sourced from the central and southern GBR exhibited decreased survival compared to southern GBR purebreds when transplanted to the southern GBR (van Oppen et al., 2014). In contrast, intraspecific hybrid offspring from far northern and central GBR populations transplanted to central reef conditions did not exhibit trade-offs in larval weight and survival, settlement competency or juvenile field survival (Quigley et al., 2016). This may be due to the relatively short period of time in the field (∼1 month) or the temperatures at the central transplant site falling within the thermal reaction norm of both populations. Both studies only transplanted in one direction and thus it is unclear if these patterns would be maintained if transplanted back to the warmer, far northern reef environments. Finally, some species of corals were not typified by high levels of local adaptation or trade-offs in growth, survival and in their response to stress (Drury et al., 2017, but see Polato et al., 2010). Understanding fitness trade-offs at each end of their thermal physiological ranges (i.e. thermal reaction norms, Roth et al., 2012) is key to fully assessing how the selective crossing of different populations with varying levels of local adaptation will influence overall reef trajectories.

A limitation of this study is the lack of cool purebred juveniles due to the extirpation of larvae from these crosses. However, because implementation of this intervention would be based on the deployment of interpopulation hybrids, and given the information provided by the cross with the cool dam, it is not completely necessary to have information on performance of purebred offspring from the cooler reef. Furthermore, different source parental colonies were used to produce the WC and CW crosses, which likely introduced additional variability into the results. As with the detected variability in survival, bleaching and growth in the warm parent crosses, this potential, but unknown variability further highlights that the selection of source material for brood stock production will be key in assuring the success of any intervention method aimed at increasing thermal tolerance in corals.

### Conclusion

The production and reseeding of cool reefs with corals that have a comparatively high thermal tolerance can facilitate adaptation to climate change. This technique has thus been proposed as a way of increasing the likelihood of successful reef-restoration initiatives (Anthony et al., 2017; Quigley et al., 2019; van Oppen et al., 2014, 2015). Despite the small sample size of reproductive colonies, this study demonstrates that crosses consisting of two parents from the warmest reef provided increased growth and survival coupled with decreased bleaching under experimentally elevated temperature in coral juveniles. The greatest overall fold-benefits in survival and growth occurred in having at least one warm dam and in symbiosis with *D. trenchii*. Encouragingly, even the use of a warm dam in combination with a cool sire provided considerable host benefits. This *ex situ* breeding of corals from warm with cool reefs may thus be used to prepare cooler reefs for further warming and summer heat waves. The next phase in the development of this intervention strategy should focus on field-based experiments to confirm these observations in the wild.

## MATERIALS AND METHODS

### Coral spawning, juvenile settlement and symbiosis establishment

Gravid *Acropora spathulata* colonies were collected from Tijou Reef (far northern GBR; 13°10′44.0″S, 143°56′54.6″E, permit G16/38488.1) and Backnumbers Reef (central GBR; 18°30′49.8″S, 147°09′10.7″E, permit G12/35236.1) between 20–24 November and 1–5 December 2017, respectively. Corals were dislodged with a hammer and chisel and maintained on board research vessels in flow-through seawater until they were returned to the Australian Institute of Marine Science National Sea Simulator Facility (via charter plane from Tijou Reef and via ship from Backnumbers Reef).

Tijou Reef was typified by on average higher temperatures compared with central Backnumbers Reef [15-year monthly average sea-surface temperature (SST)=26.4°C and 25.8°C, respectively; Fig. 1A,B], and a lower annual range in monthly SST (15-year average annual range in monthly SST=4.2°C and 5.2°C, respectively) (NASA's MODIS Aqua Global level 3 monthly daytime SSTs at a 4.6-km spatial resolution from 2002–2017; Minnett et al., 2004). Experimental conditions of 31°C were therefore hypothesized to exert less stress (Degree Heating Weeks) upon corals sourced from Tijou reef compared with Backnumbers reef (Fig. 1C). In 2016 and 2017, both reefs were impacted by bleaching [bleaching categories; 2016: 3 (Tijou), 3 (Backnumbers); 2017: 2 (Tijou), 4 (Backnumbers); Hughes et al., 2018].

Methods for spawning and the production of coral larvae and juveniles followed those outlined in (Quigley et al., 2017a). Briefly, three far northern colonies were individually crossed with three central colonies, resulting in 30 distinct familial crosses. We will hereafter refer to crosses as hybrids as defined by the nature of the intraspecific crosses (Chan et al., 2019). All 30 familial crosses were reared at 27.5°C. By the time of larval settlement, only five familial crosses remained, with all larvae from the purebred Backnumbers reef familial crosses and other cross combinations (CW and WC) were exhausted through a combination of use in larval experiments and through culture attrition. Hence, those data are not presented here. The five familial crosses focused on here include three crosses produced from parents from a warm far northern reef (WW1, WW2, WW3), one cross with a warm dam and cool sire (WC) and one cross with a cool dam and warm sire (CW) (Table 1; Table S1). Larvae were reared at a density of 1.5 larvae/ml in 15 l cone-shaped rearing tanks at 27.5°C, with 0.2 l/min flow-through seawater, resulting in one turnover per h per tank, with gentle aeration to keep larvae in the water column. These familial crosses were then settled at 27.5°C onto new (unconditioned) carbonate plugs *en masse* by adding the larvae from each cross into separate, sterilized 45-l tanks and turning off flow-through seawater for 24 h to allow larvae to settle. Once flow resumed, settled juveniles were grown on plugs for 11 days.

The numbers of plugs with settled juveniles were quantified for each familial cross and subsequently divided among three replicate tanks for symbiosis establishment at 27.5°C. Juveniles were exposed to one of three treatments of the following Symbiodiniaceae taxa cultured at the Australian Institute of Marine Science Algal Culture Facility: *S. tridacnidorum* (monoclonal SCF022.01), *C. goreaui* (monoclonal SCF055-01.10) and *D. trenchii* (heterogeneous SCF082) following Quigley et al., 2014. For symbiosis establishment, the water volume in each 45-l tank was reduced and cells from each Symbiodiniaceae type were added to each tank such that the final volume was equal to 5 l with the added volume of symbiont cells, for a final algal cell density of 1×10^5^ ml^−1^. Flow was suspended for 12 h. This procedure was repeated 2 days later, this time suspending flow for 36 h. All inoculated juveniles were subsequently kept at 27.5°C for 8 days and symbiosis establishment was visually confirmed over this period under a microscope. Juveniles were fed daily with a mix of artemia (0.5 nauplii/ml) and a mixed species microalgae recipe (10^6^ cells/ml), and were exposed to a 12:12 day:night light cycle of ∼171 PAR. Plugs were then randomly divided across treatment tanks, and half from each symbiosis-establishment treatment were placed into 31°C treatment tanks without ramping, totalling six tanks (three replicate tanks at 27.5°C and three replicate tanks at 31°C).

### Trait measurements in juveniles

Juvenile survival, bleaching and growth were assessed through image analysis. Images were taken with a Nikon D810 with a Nikon AF-S 60 mm f/2.8 G Micro ED Lens with four Ikelite DS160 Strobes. Images were taken starting on the first day of exposure to 31°C, with five time points measured and analysed at 1, 9, 35, 49 and 70 days of heat exposure. All images include a scale bar and mini coral bleaching colour-reference card (Siebeck et al., 2006). Survival was quantified for each juvenile as alive or dead. Bleaching was quantified from photographs by visually scoring juveniles using the coral bleaching colour-reference card (CoralWatch Health Score). Juveniles were scored as highly pigmented (3=D6), pale (2=D4), bleached (1=D1, translucent tissue), or dead (0, missing or bare skeleton with or without algal or cyanobacterial overgrowth) (Fig. S1). On the first day of heating, all juveniles per family were scored (mean colour score±s.e.; *S. tridacnidorum*: 2.2±0.1 to 2.4±0.1, *C. goreaui*: 2.0±0.1 to 2.1±0.1, *D. trenchii*: 2.2±0.1 to 2.5±0.1). Juveniles were also scored for colour at subsequent timepoints. Growth was determined using the ‘Area’ tool in ImageJ (Rueden et al., 2017) after calibrating each image to the scale bar.

### Statistical analyses

#### Survival

All statistical analyses were done in R (version 3.5.1, 2018-07-02) (R Core Team, 2013). Statistical tests for all traits were assessed at two levels of host genetic background: by familial cross (WW1, WW2, WW3, WC, CW) and by the geographical source of the parental corals (WW, WC, CW). Survival was assessed using a generalized linear model, fit with a binomial distribution (alive or dead) and included the interactive fixed effects of symbiont identity, host genetic background (e.g. familial cross or parental source location), and temperature treatment, with time (five factorial levels for each timepoint) and replicate tanks (six factorial levels in which three are within each temperature treatment) set as random effects blocking factors using the ‘MASS’ and ‘nlme’ packages (Pinheiro et al., 2014; Venables and Ripley, 2002). Kaplan–Meier Survival curves and associated *P*-values were calculated using the survfit function from the ‘Survival’ package (Therneau, 2015). Tukey post-hoc tests were performed using the package ‘lsmeans’ (Lenth and Hervé, 2015). Assumptions of normality and homogeneity of variances were assessed with the ‘sjPlot’ package (Lüdecke, 2017). No auto-correlation patterns were detected in the residuals. The relative contributions of symbiont identity and host genetic background were quantified using statistical methods in which each factor was run separately as described in (Mizerek et al., 2018) using Marginal and Conditional R^2^ values calculated with the ‘rsquared’ function from the ‘piecewiseSEM’ package (Lefcheck, 2016).

#### Bleaching and growth

Percent change in the bleaching score and juvenile area were calculated for each individual juvenile across host genetic background and symbiont type. Percent change was calculated between the first and last timepoint {[(Time_final_−Time_initial_)/Time_initial_]×100}. Percent change was used given that the initial level of symbiosis establishment and/or pigmentation varied slightly by symbiont type (see ‘Trait measurements in juveniles’ above for details).

Generalized linear mixed effect models with Gaussian distributions were run using the ‘glmer’ function from the package ‘lme4’ (Bates et al., 2014) to assess the relative contribution of symbiont identity and host genetic background (at either the familial cross or parental source location level) in explaining bleaching variability. Symbiont identity, host genetic background, temperature and the pairwise interactions for all three factors were treated as fixed effects. Replicate tanks were treated as a random effect. All statistical analyses followed information given above for survival.

Corals exhibit a wide range of phenotypic variability in their responses to thermal stress, where some individuals may die outright without bleaching whereas others will bleach heavily but not die (McClanahan, 2004; Tchernov et al., 2011). Given this variability and the decoupling between phenotypes associated with bleaching, mortality, and potentially growth, models were run both with (dead juveniles as ‘zeros’) and without juveniles (‘zeros’ removed) that had died by the final timepoint. Results are therefore discussed in terms of ‘bleaching and death’ or ‘growth and death’ (both including dead juveniles as zeros) compared to ‘bleaching’ or ‘growth’ of survivors only (dead juveniles excluded from the analyses). Both analyses are included to demonstrate overall trends within groups (bleaching and mortality or growth and mortality), as well as trends within only those juveniles that survived.

To assess the effect of genetic and symbiont identity on the percentage change in growth of surviving juveniles, a Gaussian mixed effects model in a Bayesian framework utilising the package ‘MCMCglmm’ was used (nitt=50,000; burnin=10,000; thin=20) (Hadfield, 2010). The interaction of symbiont by host genetic identity was set as the fixed factor, with replicate tanks treated as a random effect. Percentage change in area was assessed using the same model construction as described above. The ‘lsmeans’ package was used to extract relevant comparisons. Assumptions of chain mixing, posterior distribution normality and lack of autocorrelation were met. The relative contributions of symbiont identity and host genetic background were quantified from MCMCglmm models from manually calculated Marginal and Conditional R^2^ values.

All treatment combinations (temperature×Symbiodiniaceae treatment×familial cross) had greater than five replicate individuals for statistical analysis, although in 6 out of the 30 combinations, these individuals were distributed over only two and not all three replicate tanks due to mortality of juveniles. To quantify if the lack of three tank replicates in a limited number of treatment combinations (6 of 30) impacted model outcomes, we tested for the influence of tank effects using linear mixed models (fixed: temperature×Symbiodiniaceae treatment×familial cross; random: Tank), with model selection performed with AIC and the log-likelihood ratio test using the ‘anova’ function in the ‘nlme’ package (Pinheiro and Bates, 2006). Tank effects did not significantly explain variation in bleaching status either when only surviving juveniles were considered (LME: *P=*0.95, AIC=1327.3 versus 1325.3) or when dead juveniles counted as zeros were included (LME: *P=*0.05, AIC=4186.4 versus 4188.2).

### Relative importance of genetic identity and symbiont for survival, growth and bleaching

The odds of survival, bleaching and growth were estimated by calculating the proportion of variance attributed to symbiont identity, host genetic background and their interaction (Dixon et al., 2015). A model incorporating the interactive effects of genetic and symbiont identity was fit, as described above, with the random effects of time and replicate tanks averaged across both temperature treatments. Models were fit using global intercept contrast coding such that each treatment (familial cross×symbiont combination) was compared to the global mean of all treatments (i.e. the mean of all other treatments together). The relative importance (proportion of variance) of genetic identity and symbiont interactions were estimated as described above.

To assess the effect of genetic and symbiont identity on the percentage change in bleaching level of surviving juveniles, a Bayesian Gaussian mixed effects model was used (nitt=50,000; burnin=10,000; thin=20) (Hadfield, 2010). The interaction of symbiont by host genetic identity was set as the fixed factor, with replicate tanks treated as a random effect. Percentage change in area was assessed using the same model construction as described above.

## Supplementary Material

Supplementary information

## References

[BIO047316C1] Abrego D., Ulstrup K. E., Willis B. L. and van Oppen M. J. H. (2008). Species–specific interactions between algal endosymbionts and coral hosts define their bleaching response to heat and light stress. *Proc. R. Soc. B* 275, 2273-2282. 10.1098/rspb.2008.0180 PMC2603234 18577506

[BIO047316C3] Anthony K., Bay L. K., Costanza R., Firn J., Gunn J., Harrison P., Heyward A., Lundgren P., Mead D. and Moore T. (2017). New interventions are needed to save coral reefs. *Nat. Ecol. Evol.* 1, 1420 10.1038/s41559-017-0313-5 29185526

[BIO047316C4] Baird A. H., Bhagooli R., Ralph P. J. and Takahashi S. (2009). Coral bleaching: the role of the host. *Trends Ecol. Evol.* 24, 16-20. 10.1016/j.tree.2008.09.005 19022522

[BIO047316C5] Baker A. C., Starger C. J., McClanahan T. R. and Glynn P. W. (2004). Coral reefs: corals' adaptive response to climate change. *Nature* 430, 741 10.1038/430741a 15306799

[BIO047316C6] Bates D., Maechler M., Bolker B. and Walker S. (2014). lme4: Linear mixed-effects models using Eigen and S4 *R Packag. version* 1.

[BIO047316C7] Berkelmans R. and van Oppen M. J. H. (2006). The role of zooxanthellae in the thermal tolerance of corals: a ‘nugget of hope’ for coral reefs in an era of climate change. *Proc. R. Soc. B Biol. Sci.* 273, 2305-2312. 10.1098/rspb.2006.3567 PMC1636081 16928632

[BIO047316C8] Bucher M., Wolfowicz I., Voss P. A., Hambleton E. A. and Guse A. (2016). Development and symbiosis establishment in the cnidarian endosymbiosis model *Aiptasia* sp. *Sci. Rep.* 6, 19867 10.1038/srep19867 26804034 PMC4726165

[BIO047316C9] Cantin N. E., van Oppen M. J. H., Willis B. L., Mieog J. and Negri A. P. (2009). Juvenile corals can acquire more carbon from high-performance algal symbionts. *Coral Reefs* 28, 405-414. 10.1007/s00338-009-0478-8

[BIO047316C10] Chan W. Y., Hoffmann A. A. and van Oppen M. J. (2019). Hybridization as a conservation management tool. *Conserv. Lett.* 12, e12652 10.1111/conl.12652

[BIO047316C11] Császár N. B. M., Seneca F. O. and van Oppen M. J. H. (2009). Variation in antioxidant gene expression in the scleractinian coral *Acropora millepora* under laboratory thermal stress. *Mar. Ecol. Prog. Ser.* 392, 93-102. 10.3354/meps08194

[BIO047316C12] Császár N. B. M., Ralph P. J., Frankham R., Berkelmans R. and van Oppen M. J. H. (2010). Estimating the potential for adaptation of corals to climate warming. *PLoS ONE* 5, e9751 10.1371/journal.pone.0009751 20305781 PMC2841186

[BIO047316C13] Cumbo V. R., van Oppen M. J. H. and Baird A. H. (2018). Temperature and Symbiodinium physiology affect the establishment and development of symbiosis in corals. *Mar. Ecol. Prog. Ser.* 587, 117-127. 10.3354/meps12441

[BIO047316C81] Cunning R. and Baker A. (2014). Not just who, but how many: the importance of partner abundance in reef coral symbioses. *Frontiers in Microbology* 5, 400 10.3389/fmicb.2014.00400 PMC4120693 25136339

[BIO047316C14] Cunning R., Gillette P., Capo T., Galvez K. and Baker A. C. (2015). Growth tradeoffs associated with thermotolerant symbionts in the coral *Pocillopora damicornis* are lost in warmer oceans. *Coral Reefs* 34, 155-160. 10.1007/s00338-014-1216-4

[BIO047316C15] De'ath G., Fabricius K. E., Sweatman H. and Puotinen M. (2012). The 27–year decline of coral cover on the Great Barrier Reef and its causes. *Proc. Natl. Acad. Sci. USA* 109, 17995-17999. 10.1073/pnas.1208909109 23027961 PMC3497744

[BIO047316C16] Dixon G. B., Davies S. W., Aglyamova G. V., Meyer E., Bay L. K. and Matz M. V. (2015). Genomic determinants of coral heat tolerance across latitudes. *Science* 348, 1460-1462. 10.1126/science.1261224 26113720

[BIO047316C17] Drury C., Manzello D. and Lirman D. (2017). Genotype and local environment dynamically influence growth, disturbance response and survivorship in the threatened coral, *Acropora cervicornis*. *PLoS ONE* 12, e0174000 10.1371/journal.pone.0174000 28319134 PMC5358778

[BIO047316C18] Hadfield J. D. (2010). MCMC methods for multi-response generalized linear mixed models: the MCMCglmm R package. *J. Stat. Softw.* 33, 1-22. 10.18637/jss.v033.i0220808728

[BIO047316C19] Hereford J. (2009). A quantitative survey of local adaptation and fitness trade-offs. *Am. Nat.* 173, 579-588. 10.1086/597611 19272016

[BIO047316C20] Heron S. F., Maynard J. A. and Ruben van Hooidonk C. (2016). Warming trends and bleaching stress of the World's coral reefs 1985-2012. *Sci. Rep.* 6, 38402 10.1038/srep38402 27922080 PMC5138844

[BIO047316C21] Hoadley K. D., Lewis A. M., Wham D. C., Pettay D. T., Grasso C., Smith R., Kemp D. W., LaJeunesse T. C. and Warner M. E. (2019). Host–symbiont combinations dictate the photo-physiological response of reef-building corals to thermal stress. *Sci. Rep.* 9, 9985 10.1038/s41598-019-46412-4 31292499 PMC6620294

[BIO047316C22] Howells E. J., Beltran V. H., Larsen N. W., Bay L. K., Willis B. L. and van Oppen M. J. H. (2012). Coral thermal tolerance shaped by local adaptation of photosymbionts. *Nat. Clim. Change* 2, 116-120. 10.1038/nclimate1330

[BIO047316C23] Howells E. J., Berkelmans R., van Oppen M. J. H., Willis B. L. and Bay L. K. (2013). Historical thermal regimes define limits to coral acclimatization. *Ecology* 94, 1078-1088. 10.1890/12-1257.1 23858648

[BIO047316C24] Hughes T. P., Kerry J. T., Álvarez-Noriega M., Álvarez-Romero J. G., Anderson K. D., Baird A. H., Babcock R. C., Beger M., Bellwood D. R., Berkelmans R. et al. (2017). Global warming and recurrent mass bleaching of corals. *Nature* 543, 373-377. 10.1038/nature21707 28300113

[BIO047316C25] Hughes T. P., Anderson K. D., Connolly S. R., Heron S. F., Kerry J. T., Lough J. M., Baird A. H., Baum J. K., Berumen M. L., Bridge T. C. et al. (2018). Spatial and temporal patterns of mass bleaching of corals in the Anthropocene. *Science* 359, 80-83. 10.1126/science.aan8048 29302011

[BIO047316C26] Jin Y. K., Lundgren P., Lutz A., Raina J.-B., Howells E. J., Paley A. S., Willis B. L. and van Oppen M. J. H. (2016). Genetic markers for antioxidant capacity in a reef-building coral. *Sci. Adv.* 2, e1500842 10.1126/sciadv.1500842 27386515 PMC4928996

[BIO047316C27] Jones A. and Berkelmans R. (2010). Potential costs of acclimatization to a warmer climate: growth of a reef coral with heat tolerant vs. sensitive symbiont types. *PLoS ONE* 5, e10437 10.1371/journal.pone.0010437 20454653 PMC2862701

[BIO047316C28] Jones A. M. and Berkelmans R. (2011). Tradeoffs to thermal acclimation: energetics and reproduction of a reef coral with heat tolerant *Symbiodinium* type-D. *J. Mar. Biol.* 2011, 185890 10.1155/2011/185890

[BIO047316C29] Kenkel C. D., Goodbody-Gringley G., Caillaud D., Davies S. W., Bartels E. and Matz M. V. (2013). Evidence for a host role in thermotolerance divergence between populations of the mustard hill coral *(Porites astreoides)* from different reef environments. *Mol. Ecol.* 22, 4335-4348. 10.1111/mec.12391 23906315

[BIO047316C30] Kenkel C. D., Almanza A. T. and Matz M. V. (2015a). Fine-scale environmental specialization of reef-building corals might be limiting reef recovery in the Florida Keys. *Ecology* 96, 3197-3212. 10.1890/14-2297.1 26909426

[BIO047316C31] Kenkel C. D., Setta S. P. and Matz M. V. (2015b). Heritable differences in fitness-related traits among populations of the mustard hill coral, *Porites astreoides*. *Heredity (Edinb).* 115, 509-516. 10.1038/hdy.2015.52 26081798 PMC4806898

[BIO047316C32] LaJeunesse T. L. (2002). Diversity and community structure of symbiotic dinoflagellates from Caribbean coral reefs. *Mar. Biol.* 141, 387-400. 10.1007/s00227-002-0829-2

[BIO047316C33] LaJeunesse T. C., Parkinson J. E., Gabrielson P. W., Jeong H. J., Reimer J. D., Voolstra C. R. and Santos S. R. (2018). Systematic revision of Symbiodiniaceae highlights the antiquity and diversity of coral endosymbionts. *Curr. Biol.* 28, 2570-2580.e6. 10.1016/j.cub.2018.07.008 30100341

[BIO047316C34] Lefcheck J. S. (2016). piecewiseSEM: piecewise structural equation modelling in r for ecology, evolution, and systematics. *Methods Ecol. Evol.* 7, 573-579. 10.1111/2041-210X.12512

[BIO047316C35] Lenth R. V. and Hervé M. (2015). Package ‘lsmeans’ lsmeans: Least-squares means R package version 2.19.

[BIO047316C36] Little A. F., van Oppen M. J. H. and Willis B. L. (2004). Flexibility in algal endosymbioses shapes growth in reef corals. *Science* 304, 1492-1494. 10.1126/science.1095733 15178799

[BIO047316C37] Lüdecke D. (2017). sjPlot: Data Visualization for Statistics in Social Science R Package Version 2.3. 3.

[BIO047316C38] Manzello D. P., Matz M. V., Enochs I. C., Valentino L., Carlton R. D., Kolodziej G., Serrano X., Towle E. K. and Jankulak M. (2019). Role of host genetics and heat-tolerant algal symbionts in sustaining populations of the endangered coral Orbicella faveolata in the Florida Keys with ocean warming. *Glob. Change Biol.* 25, 1016-1031. 10.1111/gcb.14545 30552831

[BIO047316C39] McClanahan T. R. (2004). The relationship between bleaching and mortality of common corals. *Mar. Biol.* 144, 1239-1245. 10.1007/s00227-003-1271-9

[BIO047316C40] McIlroy S. E. and Coffroth M. A. (2017). Coral ontogeny affects early symbiont acquisition in laboratory-reared recruits. *Coral Reefs* 36, 927-932. 10.1007/s00338-017-1584-7

[BIO047316C41] McIlroy S. E., Gillette P., Cunning R., Klueter A., Capo T., Baker A. C. and Coffroth M. A. (2016). The effects of *Symbiodinium (Pyrrhophyta)* identity on growth, survivorship, and thermal tolerance of newly settled coral recruits. *J. Phycol.* 52, 1114-1124. 10.1111/jpy.12471 27690269

[BIO047316C42] Mieog J. C., Olsen J. L., Berkelmans R., Bleuler-Martinez S. A., Willis B. L. and van Oppen M. J. H. (2009). The roles and interactions of symbiont, host and environment in defining coral fitness. *PLoS ONE* 4, e6364 10.1371/journal.pone.0006364 19629182 PMC2710517

[BIO047316C43] Mies M., Güth A. Z., Castro C. B., Pires D. O., Calderon E. N., Pompeu M. and Sumida P. Y. G. (2018). Bleaching in reef invertebrate larvae associated with *Symbiodinium* strains within clades A–F. *Mar. Biol.* 165, 6 10.1007/s00227-017-3263-1

[BIO047316C44] Minnett P. J., Brown O. B., Evans R. H., Key E. L., Kearns E. J., Kilpatrick K., Kumar A., Maillet K. A. and Szczodrak G. (2004). Sea-surface temperature measurements from the Moderate-Resolution Imaging Spectroradiometer (MODIS) on Aqua and Terra. In IGARSS 2004. 2004 IEEE International Geoscience and Remote Sensing Symposium, 4576-4579.

[BIO047316C45] Mizerek T. L., Baird A. H. and Madin J. S. (2018). Species traits as indicators of coral bleaching. *Coral Reefs* 37, 791-800. 10.1007/s00338-018-1702-1

[BIO047316C46] Moberg F. and Folke C. (1999). Ecological goods and services of coral reef ecosystems. *Ecol. Econ.* 29, 215-233. 10.1016/S0921-8009(99)00009-9

[BIO047316C47] Mohamed A. R., Cumbo V., Harii S., Shinzato C., Chan C. X., Ragan M. A., Bourne D. G., Willis B. L., Ball E. E. and Satoh N. (2016). The transcriptomic response of the coral *Acropora digitifera* to a competent *Symbiodinium* strain: the symbiosome as an arrested early phagosome. *Mol. Ecol.* 25, 3127-3141. 10.1111/mec.13659 27094992

[BIO047316C48] O'Mahoney J., Simes R., Redhill D., Heaton K., Atkinson C., Hayward E. and Nguyen M. (2017). At what price? The economic, social and icon value of the Great Barrier Reef. Brisbane: Deloitte Access Economics.

[BIO047316C49] Ortiz J. C., González-Rivero M. and Mumby P. J. (2013). Can a thermally tolerant symbiont improve the future of Caribbean coral reefs? *Glob. Change Biol.* 19, 273-281. 10.1111/gcb.12027 23504738

[BIO047316C50] Palumbi S. R., Barshis D. J., Traylor-Knowles N. and Bay R. A. (2014). Mechanisms of reef coral resistance to future climate change. *Science* 344, 895-898. 10.1126/science.1251336 24762535

[BIO047316C51] Parkinson J. E. and Baums I. B. (2014). The extended phenotypes of marine symbioses: ecological and evolutionary consequences of intraspecific genetic diversity in coral-algal associations. *Front. Microbiol.* 5, 445 10.3389/fmicb.2014.00445 25202306 PMC4142987

[BIO047316C52] Pettay D. T., Wham D. C., Smith R. T., Iglesias-Prieto R. and LaJeunesse T. C. (2015). Microbial invasion of the Caribbean by an Indo-Pacific coral zooxanthella. *Proc. Natl. Acad. Sci. USA* 112, 7513-7518. 10.1073/pnas.1502283112 26034268 PMC4475936

[BIO047316C53] Pinheiro J. and Bates D. (2006). *Mixed-Effects Models in S and S-PLUS*. Springer Science & Business Media.

[BIO047316C54] Pinheiro J., Bates D., DebRoy S. and Sarkar D. (2014). Nlme: linear and nonlinear mixed effects models R package version 3.1-118.

[BIO047316C55] Polato N. R., Voolstra C. R., Schnetzer J., DeSalvo M. K., Randall C. J., Szmant A. M., Medina M. and Baums I. B. (2010). Location-specific responses to thermal stress in larvae of the reef-building coral *Montastraea faveolata*. *PLoS ONE* 5, e11221 10.1371/journal.pone.0011221 20585643 PMC2890407

[BIO047316C56] Quigley K. M., Davies S. W., Kenkel C. D., Willis B. L., Matz M. V. and Bay L. K. (2014). Deep-sequencing method for quantifying background abundances of *Symbiodinium* types: exploring the rare *Symbiodinium* biosphere in reef-building corals. *PLoS ONE* 9, e94297 10.1371/journal.pone.0094297 24728373 PMC3984134

[BIO047316C57] Quigley K. M., Willis B. L. and Bay L. K. (2016). Maternal effects and *Symbiodinium* community composition drive differential patterns in juvenile survival in the coral *Acropora tenuis*. *R. Soc. open sci.* 3, 160471 10.1098/rsos.160471 27853562 PMC5098987

[BIO047316C58] Quigley K. M., Willis B. L. and Bay L. K. (2017a). Heritability of the *Symbiodinium* community in vertically-and horizontally-transmitting broadcast spawning corals. *Sci. Rep.* 7, 8219 10.1038/s41598-017-08179-4 28811517 PMC5557748

[BIO047316C59] Quigley K. M., Bay L. K. and Willis B. L. (2017b). Temperature and water quality-related patterns in sediment-associated *Symbiodinium* communities impact symbiont uptake and fitness of juveniles in the genus Acropora. *Front. Mar. Sci.* 4, 401 10.3389/fmars.2017.00401

[BIO047316C60] Quigley K. M., Baker A. C., Coffroth M. A., Willis B. L. and van Oppen M. J. H. (2018). Bleaching Resistance and the Role of Algal Endosymbionts. In *Coral Bleaching* (M. J. H. van Oppen, J. M. Lough), pp. 111-151. Springer.

[BIO047316C61] Quigley K. M., Bay L. K. and van Oppen M. J. H. (2019). The active spread of adaptive variation for reef resilience. *Ecol. Evol.* 9, 11122-11135. 10.1002/ece3.5616 31641460 PMC6802068

[BIO047316C62] R Core Team (2013). *R: A language and Environment for Statistical Computing*. Vienna, Austria: R Foundation for Statistical Computing.

[BIO047316C63] Rohwer F., Seguritan V., Azam F. and Knowlton N. (2002). Diversity and distribution of coral-associated bacteria. *Mar. Ecol. Prog. Ser.* 243, 1-10. 10.3354/meps243001

[BIO047316C64] Roth M. S., Goericke R. and Deheyn D. D. (2012). Cold induces acute stress but heat is ultimately more deleterious for the reef-building coral Acropora yongei. *Sci. Rep.* 2, 240 10.1038/srep00240 22355753 PMC3270498

[BIO047316C65] Rueden C. T., Schindelin J., Hiner M. C., DeZonia B. E., Walter A. E., Arena E. T. and Eliceiri K. W. (2017). ImageJ2: ImageJ for the next generation of scientific image data. *BMC Bioinformatics* 18, 529 10.1186/s12859-017-1934-z 29187165 PMC5708080

[BIO047316C66] Siebeck U. E., Marshall N. J., Klüter A. and Hoegh-Guldberg O. (2006). Monitoring coral bleaching using a colour reference card. *Coral Reefs* 25, 453-460. 10.1007/s00338-006-0123-8

[BIO047316C67] Stat M. and Gates R. D. (2011). Clade D *Symbiodinium* in Scleractinian corals: a “nugget” of hope, a selfish opportunist, an ominous sign, or all of the above? *J. Mar. Biol.* 2011, 1-9. 10.1155/2011/730715

[BIO047316C68] Suzuki G., Yamashita H., Kai S., Hayashibara T., Suzuki K., Iehisa Y., Okada W., Ando W. and Komori T. (2013). Early uptake of specific symbionts enhances the post-settlement survival of Acropora corals. *Mar. Ecol. Prog. Ser.* 494, 149-158. 10.3354/meps10548

[BIO047316C69] Tchernov D., Kvitt H., Haramaty L., Bibby T. S., Gorbunov M. Y., Rosenfeld H. and Falkowski P. G. (2011). Apoptosis and the selective survival of host animals following thermal bleaching in zooxanthellate corals. *Proc. Natl. Acad. Sci. USA* 108, 9905-9909. 10.1073/pnas.1106924108 21636790 PMC3116386

[BIO047316C70] Therneau T. (2015). A Package for Survival Analysis in S. version 2.38.

[BIO047316C71] Thompson D. M. and Van Woesik R. (2009). Corals escape bleaching in regions that recently and historically experienced frequent thermal stress. *Proc. R. Soc. Lond. B Biol. Sci.* 276, 2893-2901. 10.1098/rspb.2009.0591 PMC2817205 19474044

[BIO047316C72] van Oppen M. J. H., Palstra F. P., Piquet A. M.-T. and Miller D. J. (2001). Patterns of coral–dinoflagellate associations in Acropora: significance of local availability and physiology of *Symbiodinium* strains and host–symbiont selectivity. *Proc. R. Soc. Lond. Ser. B Biol. Sci.* 268, 1759-1767. 10.1098/rspb.2001.1733 PMC1088806 11522193

[BIO047316C73] van Oppen M. J. H., Puill-Stephan E., Lundgren P., De'ath G. and Bay L. K. (2014). First-generation fitness consequences of interpopulational hybridisation in a Great Barrier Reef coral and its implications for assisted migration management. *Coral Reefs* 33, 607-611. 10.1007/s00338-014-1145-2

[BIO047316C74] van Oppen M. J. H., Oliver J. K., Putnam H. M. and Gates R. D. (2015). Building coral reef resilience through assisted evolution. *Proc. Natl. Acad. Sci. USA* 112, 2307-2313. 10.1073/pnas.1422301112 25646461 PMC4345611

[BIO047316C75] Venables W. N. and Ripley B. D. (2002). Modern Applied Statistics with S.

[BIO047316C76] Yamashita H., Suzuki G., Kai S., Hayashibara T. and Koike K. (2014). Establishment of coral-algal symbiosis requires attraction and selection. *PLoS ONE* 9, e97003 10.1371/journal.pone.0097003 24824794 PMC4019531

[BIO047316C77] Yorifuji M., Harii S., Nakamura R. and Fudo M. (2017). Shift of symbiont communities in *Acropora tenuis* juveniles under heat stress. *PeerJ* 5, e4055 10.7717/peerj.4055 29255647 PMC5732543

[BIO047316C78] Young C. N., Schopmeyer S. A. and Lirman D. (2012). A review of reef restoration and coral propagation using the threatened genus Acropora in the Caribbean and Western Atlantic. *Bull. Mar. Sci.* 88, 1075-1098. 10.5343/bms.2011.1143

[BIO047316C79] Yuyama I. and Higuchi T. (2014). Comparing the effects of symbiotic algae (*Symbiodinium*) clades C1 and D on early growth stages of *Acropora tenuis*. *PLoS ONE* 9, e98999 10.1371/journal.pone.0098999 24914677 PMC4051649

[BIO047316C80] Yuyama I., Nakamura T., Higuchi T. and Hidaka M. (2016). Different stress tolerances of juveniles of the coral *Acropora tenuis* associated with clades C1 and D *Symbiodinium*. *Zool. Stud.* 55, 1-9. 10.6620/ZS.2016.55-19 PMC6409504 31966164

