# Supplementary material for: Assessing the role of historical temperature regime and algal symbionts on the heat tolerance of coral juveniles
Source: Biol Open. 2020 Jan 23;9(1):bio047316. doi: 10.1242/bio.047316 (PMC6994947; doi:10.1242/bio.047316)
Supplement: Supplementary information [file biolopen-9-047316-s1.pdf]

Supplementary Material

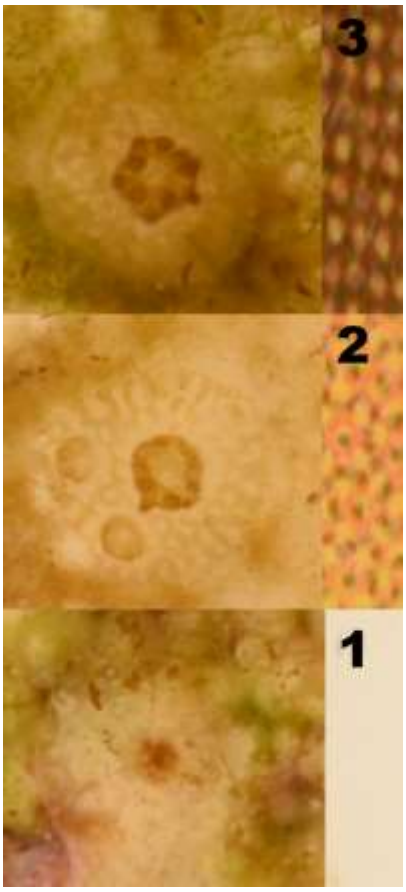

Figure S1. Juvenile photos compared to coral bleaching colour reference card (Siebeck et al. 2006). Bleaching was quantified from images by visually scoring whole juveniles using the coral Bleaching Colour Reference Card. Juveniles were scored as highly pigmented ("3" = D6), pale ("2" = D4), bleached ("1" = D1, translucent tissue), or dead (0). Photographs here are all juveniles from the first day at 31°C (first timepoint) and from the *S. tridacnidorum* treatment, after 11 days post initial symbiont exposure.

Table S1. Sample sizes of juveniles for each treatment at the initial timepoint (1 day).

	C. goreaui		*S. tridacnidorum*		*D. trenchii*	
	27°C	32°C	27°C	32°C	27°C	32°C
WW1	14	16	15	8	29	24
WW2	32	14	16	11	25	29
WW3	32	21	8	7	15	19
WC	16	6	4	7	8	8
CW	11	8	6	7	6	5
